# An internet-based behavioral intervention for adults with autism spectrum disorder – A randomized controlled trial and feasibility study

**DOI:** 10.1016/j.invent.2023.100672

**Published:** 2023-09-19

**Authors:** Britta Westerberg, Fredrik Holländare, Susanne Bejerot

**Affiliations:** a University Health Care Research Center, Faculty of Medicine and Health, Örebro University, Örebro, Sweden; b Department of Psychiatry, Faculty of Medicine and Health, Örebro University, Örebro, Sweden; c School of Medical Sciences, Faculty of Medicine and Health, Örebro University, Örebro, Sweden

**Keywords:** Autism spectrum disorder, Internet-based intervention, Cognitive-behavioral therapy, Quality of life, Psychiatry

## Abstract

**Background:**

The increased prevalence of Autism Spectrum Disorder (ASD) diagnoses in combination with psychiatric comorbidity, has led to an increased need for effective interventions. The evidence for internet-based interventions for several mental health problems is established but has not been evaluated for adults with ASD.

**Objective:**

The aim of this randomized controlled trial is to evaluate the feasibility and effects of an internet-based intervention targeting quality of life and psychiatric symptoms (depression and anxiety) in adults with ASD.

**Methods:**

84 participants were randomly allocated to intervention (*n* = 42) or control (n = 42). The 18-week internet-based intervention covered a range of themes related to difficulties common in ASD, and exercises based on cognitive behavioral strategies. Participants were provided with individual feedback following each module and were invited to regular chat sessions with peer participants. The primary outcomes were subjective quality of life and sense of coherence, and secondary outcomes were symptoms of depression and anxiety. All outcomes were measured at five occasions and analysed with linear mixed effect models. Participant satisfaction and adherence was also analysed.

**Results:**

Participant satisfaction and adherence was satisfactory but no significant interaction between group and time was found for any outcome measure. Autistic traits were negatively related to quality of life and sense of coherence and positively related to anxiety and depressive symptoms.

**Conclusions:**

This internet-based intervention showed feasibility regarding adherence and participant satisfaction. However, no significant effects on quality of life, sense of coherence or psychiatric symptoms were found, likely due to limitations in the design and methodology of this specific trial in combination to the heterogeneity of the group. Individuals with ASD may require interventions that are flexible and individually tailored in regard to both format, content and therapeutic support. The current trial provides useful information and suggestions for the future research on internet-based interventions for ASD.

## Highlights

• Individuals diagnosed with autism have highly varying health care needs.
• Internet-based interventions are feasible among individuals with autism.
• Group chat sessions were integrated in the individualized internet-based treatment.

## 1 Introduction

Autism spectrum disorder (ASD) is a neurodevelopmental disorder that may cause extensive adaptive disabilities. Internationally, ASD diagnoses have increased substantially in prevalence over the last years (Mottron and Bzdok, 2020; Lundström et al., 2015; Fuentes et al., 2021). The Diagnostic and Statistical Manual of Mental Disorders 5 (DSM-5) defines ASD as characterized by 1: deficits in the ability for social interaction and communication and 2: repetitive patterns of interest, behavior, and activities (American Psychiatric Association, 2013). Psychiatric comorbidity is common (Murray et al., 2019; Hollocks et al., 2019; Lever and Geurts, 2016; Hossain et al., 2020; Gillberg et al., 2016) and experiences of bullying are frequent (Cappadocia et al., 2012; Weiss and Fardella, 2018), both substantially affecting the Quality of Life (QOL) (Mason et al., 2018; Lord et al., 2020; van Heijst and Geurts, 2015) and may contribute to suicidality (Hirvikoski et al., 2016). The health care costs for individuals with ASD are high (Buescher et al., 2014), and many experience barriers to utilizing, accessing, or even seeking psychiatric care (Mason et al., 2019). Fear of stigma, geographical distance, and traveling difficulties are such barriers, in addition to a lack of adapted interventions and knowledge among professionals.

Psychological interventions adapted to the needs of autistic individuals include Cognitive Behavioral Therapy (CBT) for co-occurring depression (McGillivray and Evert, 2014) and anxiety disorders (Bemmer et al., 2021; Wise et al., 2019; Russell et al., 2013). Group-based social skills training has been shown to be effective for children and adolescents with ASD (Wolstencroft et al., 2018; Gates et al., 2017; Choque Olsson et al., 2017). Group-based interventions offer opportunities for peer support and a safe skill-training environment and are, therefore, recommended (Atkinson-Jones and Hewitt, 2019; Spain and Blainey, 2015). Other treatment adaptations to ASD include the use of visual illustrations and technological devices, simplifying or adding greater structure to exercises, and use of highly explicit strategies such as S.M.A.R.T (Specific, Measurable, Achievable, Realistic, and Time-limited) goals (Fenn and Byrne, 2013) and problem-solving strategies (Kerns et al., 2016; NICE Clinical Guidelines, No. 142, 2021).

A Swedish research group has developed and evaluated a group-based, 37-session intervention adapted to psychiatric patients with ASD. The intervention, called ALMA, is based on a comprehensive battery of information and exercises related to challenges common in ASD (Bejerot and Björnstjerna, 2019). An open RCT of ALMA indicated increased QOL (Cohen's d = 0.43) with longitudinal stability. Further, 88 % showed improved self-understanding, 72 % reported higher self-acceptance, and 67 % experienced increased well-being (Hesselmark et al., 2014).

While effective Internet-based interventions (IBI) increase in scope for psychiatric diagnoses such as depression (Wright et al., 2019; Furukawa et al., 2021), social anxiety (Hedman-Lagerlöf et al., 2023), Generalized Anxiety Disorder (GAD) (Eilert et al., 2021) and Attention Deficit Hyperactivity Disorder (ADHD) (Kenter et al., 2023), the evidence of such interventions for adults with ASD is limited. However, due to the mentioned barriers to access care, interventions that reach more people with ASD are warranted. Online self-help programs targeting anxiety in ASD are suggested to be a cost-effective alternative for highly able ASD individuals (Gaigg et al., 2020). Internet-based psychoeducational and supportive interventions (Backman et al., 2018; Wentz et al., 2012) as well as IBI targeting insomnia (Georen et al., 2022) and Obsessive Compulsive Disorder (OCD) (Wickberg et al., 2022), have been evaluated with good feasibility and promising results in adult ASD.

There is a lack of studies evaluating the effects on QOL from internet-based interventions for individuals with ASD. Within the framework of the current project, major themes and interventional components of ALMA have been converted into an internet-based format. The aim of this study is therefore to evaluate the effects of the intervention on QOL and psychiatric symptoms in individuals with ASD.

## 2 Material and methods

### 2.1 Design

This is a longitudinal, randomized, pragmatic clinical trial with a parallel two-group design, with an allocation ratio of 1:1. The intervention was conducted between February 2019 and June 2019 at the University health care research center, Örebro Region, Sweden. The study was approved by the regional ethics committee in Uppsala, Sweden (2017/392) and preregistered at clinicaltrials.gov (NCT03570372).

### 2.2 Participants and recruitment

Eligible participants were individuals diagnosed with ASD without intellectual disability, aged between 16 and 64 years. The participants were recruited through posters and advertisements, wherein information about the study was given. Potential participants applied for the study via an Internet-based platform. Digital informed consent was collected as part of the application.

Semi-structured telephone interviews with potential participants were conducted using MINI international neuropsychiatric interview 7.0 (Sheehan et al., 1997). Interviews were terminated when 84 participants were included. Exclusion criteria were reported ongoing CBT, intellectual disability, suicide risk, drug abuse, ongoing psychosis, changes in drug prescription six weeks prior to inclusion, and inability to read and understand Swedish. All participants gave consent for us to contact the health care facility that conducted the diagnostic assessment in order to confirm their diagnosis.

### 2.3 Randomization

An independent statistician generated a computer-aided block randomization. Allocation (“control” or “intervention”) was written inside opaque envelopes and numbered from 1 to 84. The envelopes were opened one by one by BW as the participants were included. Before the start of the study, the participants received a text message with information about their allocation, start date, and instructions to the online platform. Neither the participant nor the therapists or investigators were blinded at any stage of the intervention or subsequent analyses.

### 2.4 Control condition

The control group (*n* = 42) was provided with, and encouraged to read, two books focusing on different aspects of living with ASD. Both books were available online and handed as paper copies. The first book – available from the start - was psychoeducational, concerning symptoms of ASD, common difficulties in everyday living and strategies for managing difficulties (Norrö et al., 2004). However, the participants were not requested to implement any strategies or complete any exercises. The second book – available after nine weeks – was biographical, following the narratives of individuals diagnosed with ASD and struggling with different aspects of life (Nordgren, 2000).

### 2.5 Intervention

Participants in the intervention group (*n* = 42) were divided into six groups, each consisting of seven individuals. They received an 18-week intervention based on the therapeutic components of ALMA (Bejerot and Björnstjerna, 2019), however the intervention was condensed and in an internet-based setting. The intervention called MILAS constitutes 18 weekly text-based modules (equivalent to sessions), each focusing on one of five areas: 1) knowledge of ASD, 2) behavioral change, 3) social interaction and relations, 4) mental illness, and 5) everyday life strategies. For a detailed overview of the modules, including themes, strategies and exercises, see Appendix A. Exercises and strategies are based on CBT, including behavioral change, cognitive restructuring, and problem solving. MILAS aims to enhance QOL and decrease psychiatric symptoms by increasing self-acceptance, self-knowledge, and the ability to manage difficulties.

Participants completed exercises following every module and had an individual therapist following them through the modules, providing feedback after the completion of each module or exercise. Some exercises were based on reflection and designed to be completed in text on the platform (e.g. conducting a functional analysis) and some exercises were designed to be defined by the individual and completed “in real life” (e.g. the behavioral activation exercises). The participants were provided guidance in planning and initiating the exercises. However, the text-based exercises naturally allowed closer therapeutic monitoring, whereas the “in real life”- exercises were only monitored by the therapist based on the participants text-based reports and evaluations – as long as the participant didn't explicitly ask for help. In case of no reports from the exercises – the therapists sent regular reminders. The participants could contact their therapist at any time. At the start of the intervention, therapists were provided with a thorough treatment manual. One of the therapists was a registered nurse, and the other three were clinical psychologists.

Every two weeks, the participants were offered to participate in a group-based one-hour chat session together with the other participants from their group (*n* = 7). The participants could choose to be anonymous in the chat. The chat sessions were moderated by a therapist and based on open-ended questions regarding the themes of recent modules.

A previous publication on the participants' experiences with MILAS revealed an appreciation of the written format and the convenient design and emphasized the importance of the therapeutic relationship (Westerberg et al., 2021).

### 2.6 Data collection

All participants completed online self-assessment questionnaires on five occasions: pre-treatment, mid-treatment, post-treatment, and follow-up at 44 and 70 weeks post-treatment. In connection to the completion of post- and follow-up-measurements, participants were given a gift card of €28 per time point.

In addition to outcome variables, the first questionnaire included demographic information, including occupation, habitation, education, age when diagnosed, and clinical characteristics. Experiences of bullying were measured using a question on occurrence (“*Have you been bullied*?”), with responses ranging from “*Yes*” through “*No, but socially isolated*” to “*No*”.

### 2.7 Primary outcomes

#### 2.7.1 Quality of life

The Brunnsviken Brief Quality of Life (BBQ) 12-item scale was used to measure satisfaction in and importance of six different life areas (leisure time, view of life, creativity, learning, friendship, and view of oneself), forming an overall QOL score. Answers range from 0 (*Do not agree*) to 4 (*Agree totally*). The scores of two theme-related questions are multiplied and summed up to a total score ranging between 0 and 96 (higher values indicating higher QOL). The mean score on BBQ in a social anxiety sample was 39. BBQ was found to be a valid and reliable measure of subjective QOL (Lindner et al., 2016).

EQ5D -5L was used to measure health-related QOL. It contains five questions concerning mobility, self-care, usual activities, pain/discomfort, and anxiety/depression. Each area is rated from 1 (*I have no problems with this*) to 5 (*I have extreme problems with this*), creating a five-number health profile, corresponding to an index score representing the overall level of function. Index scores range from −1 (worst health imaginable) to 1 (full health). A thermometer-like Visual analogous scale (VAS) (0−100) is used in an additional question on the perceived overall health state. EQ5D-5L has shown excellent psychometric properties (Feng et al., 2021).

#### 2.7.2 Sense of coherence

The Sense of Coherence scale (SOC-13) is a 13-item questionnaire measuring the concept “sense of coherence” (SOC). SOC refers to the extent to which the world feels comprehensible and predictable, and whether the demands posed by life feel manageable and motivating. SOC is suggested to be closely related to QOL. The items include questions on how often the individual experiences a certain event or their view on life. Answers range from 1 (e.g. “*never happens*”) to 7 (e.g. “*happens very often*”) and total score ranges from 13 to 91 (higher values indicating a higher SOC) (Antonovsky, 1993). A systematic review of the properties of SOC-13 concluded it to be a psychometrically sound instrument (Eriksson and Lindstrom, 2005).

### 2.8 Secondary outcomes

#### 2.8.1 Psychiatric symptoms

The 14-item Hospital Anxiety and Depression scale (HADS) was used to assess symptoms of anxiety (7 items) and depression (7 items). The items are designed as statements on how the individual has felt for the last week, with responses ranging from 0 (e.g. *“totally”)* to 3 (e.g. *“not at all”) (*Snaith*, 2003**)*. The total subscale score may range from 0 to 21 (higher values indicating higher symptomatology). HADS has been shown to be a viable measure for assessing anxiety and depression in ASD, with mean scores of 9.2 on the anxiety subscale and 5.5 on the depression subscale (Uljarević et al., 2018).

#### 2.8.2 Treatment satisfaction

To evaluate treatment satisfaction, the Client Satisfaction Questionnaire (CSQ) was used. The CSQ consists of eight questions on different aspects related to the satisfaction of the treatment. Responses range from 1 to 4 (total score between 8 and 36), with a higher score indicating higher treatment satisfaction (Attkisson and Greenfield, 2004). The mean item score on CSQ was 2.8 (SD.0.8) in a sample of individuals with ASD who had participated in outpatient psychotherapy (Lipinski et al., 2019).

At post-treatment, seven additional questions were asked regarding self-perceived gains or personal change due to the treatment. Responses ranged from “Yes, definitely”, through “Yes, to some extent” to “No” (see Appendix B).The questions were developed for this project and have not been validated psychometrically.

### 2.9 Autistic traits

The RAADS-14 screen was used to measure autistic traits. It consists of 14 statements on the occurrence of autistic traits. Responses include four levels of occurrence from “*True Now & When Young*” through “*True Only Now*”, “*True When I Was Young*” to “*Never True*”. Total score ranges from 0 to 42 (median of 32 among Swedish individuals with ASD). RAADS-14 have good psychometric properties (Eriksson et al., 2013).

### 2.10 Statistical analysis

Sample size calculation was based on a previous study involving adults with ASD (Hesselmark et al., 2014), where they found an effect size of d = 0.43 (SD = 1.68) on self-assessed QOL. To get 80 % power to find a difference of that size (alpha level of 0.05), we needed to include 42 participants per group. If counting with a maximum dropout of 50 % (Christensen et al., 2009), we needed to include at least 84 participants. The calculations were made using PS - Power and Sample Size Calculations software.

To detect baseline differences between conditions and completers/dropouts, independent *t*-tests and chi-square homogeneity tests were conducted on demographic variables. Independent t-tests were conducted to determine differences between the conditions in outcome on all measurement occasions. Effect sizes were based on the predicted mean difference at post (Feingold, 2009). Dropout were defined as suggested by Christensen et al. (Christensen et al., 2009). Isolated missing items in BBQ (0.81 %) were handled through simple imputation of the individual median from the available data on the same item-type (importance or satisfaction) and in RAADS-14 (0.48 %) by simple imputation of the individual mean. There were no missing items in any other outcome.

We used linear mixed effect models to determine the relative change in outcome between conditions over time. Time was coded in the number of weeks as follows: 0 (pre), 9 (mid), 18 (post), 44 (follow-up I), and 70 (follow-up II). Random effects (intercept and slope whenever appropriate) were included, and an unstructured and variance components covariance structure was tested. To test for potential covariance with autistic traits, baseline RAADS-14 value was added to the models. All models were fitted with Maximum likelihood estimation, and according to the Intention to Treat-principle; data from both dropouts and completers were included. Model fit was determined based on significant decrease in the −2 log likelihood value. Data were analysed using SPSS statistics version 25 (IBM SPSS Statistics for Windows. 25.0 ed, 2017).

## 3 Results

Of the 176 individuals who applied to participate, 84 were randomized. In 75 cases, the diagnosis was actively confirmed. In nine cases, diagnostic confirmation was not retrieved because of study dropout (*n* = 4) or due to unsuccess in reaching the clinician who conducted the assessment (*n* = 5).

There were no differences in demographic characteristics between conditions at baseline. Table 1 shows the baseline demographic and clinical characteristics.

**Table 1:** Demographic characteristics at baseline.

				Group difference	
	Treatment, n = 42	Control, n = 42	Total, *n* = 84	Test statistic	p
Age, M (SD)	32.76 (9.52)	32.48 (9.36)	32.62 (9.39)	t(82) = 0.14	0.89
Gender, n (%) Male Female Other (non-binary/transsexual.)	19 (45.2) 20 (47.6) 3 (7.1)	13 (31.0) 28 (66.7) 1 (2.4)	32 (38.1) 48 (57.1) 4 (4.8)	χ2(2) = 3.46	0.18
Habitation, n (%) With partner and/or children Alone With parents Group/serviced housing Other	14 (33.3) 15 (35.7) 11 (26.2) 1 (2.4) 1 (2.4)	25 (59.5) 8 (19.0) 7 (16.7) 1 (2.4) 1 (2.4)	39 (46.4) 23 (27.4) 18 (21.4) 2 (2.4) 2 (2.4)	χ2(6) = 8.80	0.19
Education, n (%) College/university degree ASD-adapted schooling	5 (11.9) 1 (2.4)	12 (28.6) 5 (11.9)	17 (20.2) 6 (7.1)	χ2(1) = 3.61 χ2(2) = 2.88	0.060 .24
Age when diagnosed, n (%) 4–11 12–19 20–27 28–35 36–43 44–55	3 (7.1) 11 (26.2) 12 (28.6) 7 (16.7) 5 (11.9) 4 (9.6)	5 (11.9) 7 (16.7) 14 (33.3) 10 (23.8) 4 (9.5) 2 (4.8)	8 (9.5) 18 (21.4) 26 (31.0) 17 (20.2) 9 (10.9) 6 (7.2)		
Occupation, n (%) Employed Daily activities Student Unemployed Sick leave Other	6 (14.3) 3 (7.1) 4 (9.5) 12 (28.6) 8 (19.0) 9 (21.4)	10 (23.8) 6 (14.3) 6 (14.3) 6 (14.3) 11 (26.2) 2 (4.8)	16 (19.0) 9 (10.7) 10 (11.9) 18 (21.4) 19 (22.6) 11 (13.1)	χ2(6) = 10.21	0.12
Psychiatric comorbidity, n (%) Yes No Don't know	24 (57.1) 16 (38.1) 2 (4.8)	23 (54.8) 13 (31.0) 6 (14.3)	47 (56.0) 29 (34.5) 8 (9.5)	χ2(2) = 2,33	0.31
RAADS-14^a^, M (SD)	29.0 (7.4)	29.5 (9.6)	29.3 (8.5)	t(79) = −0.28	0.78
Experiences of bullying n (%) Yes No, but socially isolated No Don't know Missing	27 (64.3) 7 (16.7) 6 (14.3) 2 (4.8)	28 (66.7) 7 (16.7) 3 (7.1) 1 (2.4) 3 (7.1)	55 (65.5) 14 (16.7) 9 (10.7) 3 (3.6) 3 (3.6)	χ2(3) = 1.24	0.74

a Ritvo Autism and Asperger Diagnostic Scale-14 screen (Eriksson et al., 2013).

### 3.1 Dropout

**Fig. 1:**
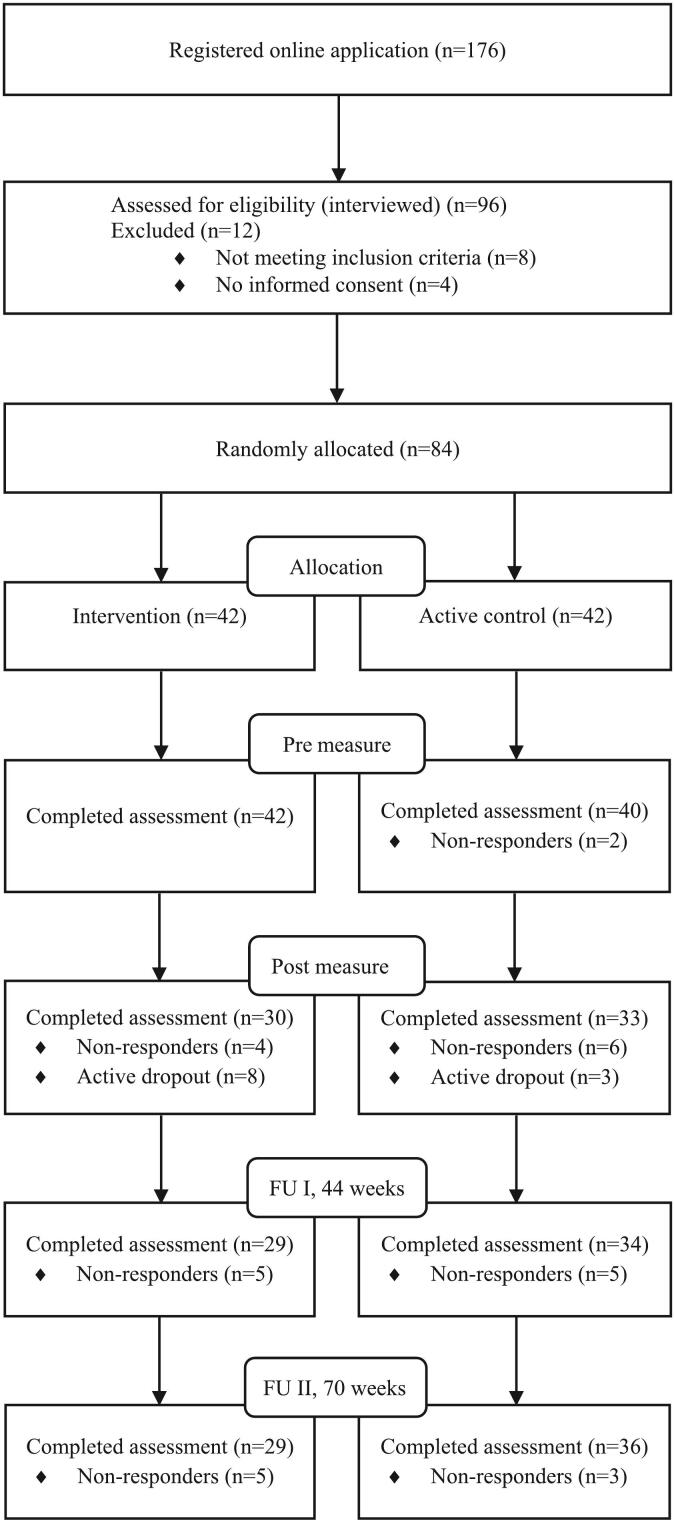
Overview of participant flow.

Eight participants (19 %) in the treatment condition and three (7 %) in the control condition actively dropped out from the study from pre to post. Reasons for dropping out of the intervention were lack of time (*n* = 2), too demanding (n = 2), somatic disease (*n* = 1), psychiatric illness (n = 2) and new medication (n = 1). The percentage of respondents was for pre: 98 %, mid: 81 %, post: 74 %, FU1: 75 %, and FU2: 77 %. An overview of participant flow is presented in Fig. 1.

Seventy-one percent of the individuals in the treatment group are labeled completers based on the completion of at least 11 of 18 modules (>60 %). Completion of a module was defined as going through the chapters and answering questions related to the theme. There was no significant difference between dropouts and completers on any demographic variable, RAADS-14, BBQ, EQ5D-5L-index, SOC or HAD. On EQ5D-5L-vas, dropouts scored lower at baseline than completers, t (40) = 2.10, *p* = 0.04.

### 3.2 Outcome analysis

Predicted means for all time points, effect size for mean difference at post and results from the conditional mixed models are shown in Table 2. There was no statistically significant mean difference between the two conditions on any outcome variable at any time point.

**Table 2:** Predicted means and test statistics^a^ for all time points, effect size (ES)^b^ for mean difference between groups at post and results from the mixed model.

		Pre measure		Post measure			Follow up I		Follow Up II		Mixed model results		
	N	M (SD)	t (df), p	M (SD)	t (df), p	ES (95 % CI)	M (SD)	t (df), p	M (SD)	t (df), p	Fixed effects	β	*p*
BBQ											Intercept	41.51	<0.001
Treatment	42	42.28 (18.43)	0.20 (81), 0.85	44.56 (18.43)	0.52 (81), 0.61	−0.10 (−0.53–0.33)	47.84 (18.43)	0.99 (81), 0.33	51.12 (18.43)	1.45 (81), 0.15	Time	0.06	0.10
Control	41	41.51 (17.61)		42.50 (17.61)			43.93 (17.61)		45.36 (17.61)		Group	0.78	0.87
											Group*time	0.07	0.15
EQ5D-5L-VAS											Intercept	61.80	<0.001
Treatment	42	57.36 (12.96)	−1.70 (81), 0.09	59.26 (12.96)	−1.24 (81), 0.22	0.14 (−0.29–0.58)	62.0 (12.96)	−0.56 (81), 0.58	64.74 (12.96)	0.11 (81), 0.91	Time	0.04	0.45
Control	41	61.80 (10.67)		62.48 (10.67)			63.46 (10.67)		63.22 (10.67)		Group	−4.44	0.30
											Group*time	0.07	0.36
EQ5D-5L-Index											Intercept	0.64	<0.001
Treatment	42	0.631 (0.157)	−0.38 (81), 0.71	0.640 (0.159)	−0.10 (81), 0.92	0.02 (−0.41–0.45)	0.653 (0.161)	0.29 (81), 0.78	0.666 (0.164)	0.65 (81), 0.52	Time	−0.00	0.93
Control	41	0.644 (0.164		0.643 (0.168)			0.642 (0.175)		0.641 (0.182)		Group	−0.01	0.77
											Group*time	0.00	0.40
SOC											Intercept	50.07	<0.001
Treatment	42	49.24 (11.40)	−0.33 (81), 0.74	50.0 (11.40)	−0.17 (81), 0.86	0.03 (−0.40–0.46)	51.05 (11.46)	0.06 (81), 0.95	52.12 (11.60)	0.28 (81), 0.78	Time	0.02	0.43
Control	41	50.07 (11.39)		50.41 (11.50)			50.90 (11.71)		51.39 (11.99)		Group	−0.84	0.77
											Group*time	0.02	0.52
HAD-anx											Intercept	7.05	<0.001
Treatment	42	11.16 (3.79)	0.92 (82), 0.36	10.86 (3.83)	0.63 (82), 0.53	−0.11 (−0.54–0.32)	10.41 (3.95)	0.22 (82), 0.83	9.97 (4.15)	−0.16 (82), 87	Time	−0.01	0.19
Control	42	10.41 (3.72)		10.33 (3.74)			10.23 (3.87)		10.12 (4.10)		Group	0.02	0.98
											Group*time	−0.01	0.40
HAD-dep											Intercept	10.41	<0.001
Treatment	42	7.07 (3.35)	0.03 (82), 0.98	6.70 (3.41)	−0.21 (82), 0.83	0.04 (−0.39–0.47)	6.17 (3.52)	−0.54 (82), 0.59	5.64 (3.65)	−0.85 (82), 0.40	Time	0.00	0.64
Control	42	7.05 (3.37)		6.86 (3.42)			6.59 (3.52)		6.32 (3.66)		Group	0.75	0.42
											Group*time	−0.01	0.34

Note: CI = Confidence interval, BBQ = Brunnsviken Brief Quality of life inventory, EQ5D-5L-VAS = EuroQol Health-related QoL questionnaire- visual analogue scale, EQ5D-5L-index = EuroQol Health-related QoL questionnaire- index, SOC = Sense Of Coherence questionnaire, HAD-anx = Hospital Anxiety and Depression Scale, anxiety, HAD-dep = Hospital Anxiety and Depression Scale, depression.

a Between group comparison with *t*-tests with a significance level of 0.05.

b Based on mean differences between groups.

#### 3.2.1 Intention to Treat analysis

EQ5D-5L-index and SOC were analysed with an unstructured covariance. Both HADS measures were analysed with a variance components structure. Only one random effect (intercept) was included for BBQ and EQ5D-VAS. A positive main effect of time on BBQ (β = 0.09, *p* ≤0.001) and a negative main effect of time on HADS-dep (β = −0.02, *p* = 0.01) were found across groups. No significant interaction between group and time was observed for any outcome.

Post hoc inclusion of baseline RAADS-14 value to the models significantly improved model fit for all outcomes, revealing a negative main effect of RAADS-14 on BBQ (β = −0.85, *p* ≤0.001) and SOC (β = −0.92, p ≤0.001) and a positive main effect of RAADS-14 on HAD-dep (β = −0.19, p ≤0.001) and HAD-anx (β = −0.25, p ≤0.001) for both groups.

#### 3.2.2 Treatment satisfaction

Treatment satisfaction according to CSQ is shown in Table 3. Based on additional questions on treatment gains, the proportion of participants who responded that they, to some extent, experience improvements are as follows: higher self-acceptance (48 %), higher understanding of their disability (62 %), more knowledge about ASD (50 %), increased self-awareness (52 %), better at expressing my needs (57 %), increased well-being (40 %), and more social contacts than before (17 %).

**Table 3:** Scores on the Client satisfaction questionnaire for the intervention group (*n* = 30).

	Mean^a^ (SD)
Total score on CSQ	25.17 (4.84)
Q1. Quality of treatment	3.10 (0.6)
Q2. The kind of service that you wanted	2.93 (0.74)
Q3. Has our program met your needs?	3.00 (0.91)
Q4. Would you recommend our program to a friend?	3.43 (0.73)
Q5. Satisfaction with the amount of help	3.20 (0.66)
Q6. Did it help you to deal with your problems?	3.07 (0.58)
Q7. General satisfaction	3.23 (0.68)
Q8. Would you come back to our program?	3.20 (0.76)

a Total score can range from 8 to 32. Individual items range from 1 to 4. Higher scores indicates higher satisfaction.

## 4 Discussion

The results show that this IBI for adults with ASD did not significantly affect QOL, SOC, or psychiatric symptoms compared to controls. However, mean item scores on CSQ reveal high levels of treatment satisfaction. In line with previous research (Mason et al., 2018; Helles et al., 2017), more autistic traits were associated with lower QOL and SOC and higher levels of depression and anxiety for both conditions.

IBI studies targeting different psychiatric conditions have had a dropout rate of between 1 and 50 % (Christensen et al., 2009). In a meta-analysis of IBI targeting depression, dropout rates between 28 and 57 % were observed for interventions with 5–12 modules (Furukawa et al., 2021). The research on IBI extending 12 weeks in length is, however, very limited; thus, a dropout of up to 50 % was expected in this trial. Based on this, the dropout of our study was, by contrast, relatively low despite the lengthy treatment, giving evidence of feasibility.

One advantage that assumedly contributed to the acceptable attrition rates might be each module's straightforward and predictable format. In the study on participant experiences, the participants did explicitly point out the convenience of the predictableness (Westerberg et al., 2021). Nevertheless, dropout rates were higher in the intervention group compared to the controls. Theoretically, the participants in the intervention group that did not experience any benefit from the treatment might have dropped out at a higher ratio. Alternatively, the dropout was higher in the intervention group simply due to higher demands on effort and engagement.

There are several potential reasons to why we were unable to detect any significant effect of the intervention. Both hindrances linked to ASD-specific difficulties and aspects related to the content and implementation of the intervention must be considered.

The treatment may have been insufficiently adapted to the individual needs of the participants. All participants were provided the same modules independently of their initially presented problems. The participants may, therefore, have perceived some modules as irrelevant to them, a theory corroborated by the findings of previous studies where lack of individual adaptations in treatment content was one of the most frequent remarks given by participants (Westerberg et al., 2021; Weisel et al., 2020). Further, in contrast to face-to-face interventions, the treatment offered no immediate support to guide through or complete exercises. Instead, therapeutic feedback was provided after every completed module, meaning that participants were expected to independently initiate and complete the tasks. According to our previous study, some participants experienced pressure completing the exercises independently (Westerberg et al., 2021), which may have resulted in an intervention group not as active as we would hope but rather avoiding the difficult exercises. As a consequence, failure to reach the specific and time-bound goals that were set, might have evoked feelings of unsuccessfulness (Oscarsson et al., 2020).

Additionally, the timeframe proposed for each module (one week) and the shortened treatment length, in contrast to the original RCT by Hesselmark et al. from 2014 (Hesselmark et al., 2014), might have been too limited, given the limited amount of therapeutic support. To be able to absorb the essential components of each theme within just one week, was likely a too high demand for many of our participants, considering the common difficulties of initiating activities and generalizing skills, as well as needs for frequent reminders and repetition.

In relation to the Hesselmark study, in which similar treatment components were provided in 2.5-h face-to-face-sessions over 37 weeks, the results of our trial are less encouraging. However, since 2014, the ASD diagnostic criteria have changed, and a rapid increase in prevalence has occurred. Today, autism is viewed as a spectrum ranging from extreme difficulties in every life area to minor deviations from normativity in fewer areas. Consequently, a phenotype that earlier did not reach thresholds for diagnosis is today labeled as a diagnosis. This heterogeneity is increasingly questioned in the scientific community (Mottron, 2021; Bejerot and Nylander, 2022) partly as it complicates research on ASD and the development of adequate interventions.

Accordingly, the participants in our study represent a heterogeneous sample of ASD individuals, including a non-prototypical subgroup with greater social awareness and resources but with a high degree of psychiatric comorbidity. As indicated by previous research (Bargiela et al., 2016; Cage and Troxell-Whitman, 2019), this phenotypical pattern is more common among females than males. Females outnumbered males in our study, also, <10 % of our sample were diagnosed ASD before puberty, findings that should be taken into consideration when reading the results. These late-diagnosed women may require other interventions (Bargiela et al., 2016), with more focus on, for example, stress management and acceptance and less focus on core skills training.

One alternative for the ASD treatment research society to manage the heterogeneity of the ASD population are either to develop fixed programs adjusted to more homogeneous subgroups of ASD individuals – i.e. one treatment for individuals suffering from much psychiatric comorbidity and another treatment for those with low social skills. Another way could be to develop individually tailored interventions in which the modules are optional and selected - in dialogue with the therapist - based on needs and motivation of the participant. This could be combined with flexibility in format, that is, offering face-to-face meetings if necessary and, for some patients, providing more practical support and prompting in planning and completing exercises.

### 4.1 Limitations

There are some limitations to the design and implementation of this study. As concluded in our previous paper (Westerberg et al., 2021), participants requested and utilized the therapeutic support to a highly varying extent, resulting in different amounts of therapeutic contact. This, combined with varying levels of therapeutic training between therapists, must be addressed as potentially important limitations. The trial would have benefitted from closer supervision and coordination between therapists. Another methodological limitation of this trial is the absence of measures targeting potential negative side effects of the intervention. However, in the qualitative study on participant experiences (Westerberg et al., 2021), negative experiences were included in the investigation, showing for example that some participants became stressed by receiving new modules before they were finished with the previous one.

Furthermore, the diagnosis was not confirmed for all participants. However, as all participants consented for us to retrieve their records and provided information on when and under which circumstances they were assessed, we have every reason to be confident that all participants had an ASD diagnosis.

The use of self-assessment questionnaires to measure subjective QOL might be a limitation in the design of the trial, considering that ASD individuals often have problems with interception and global thinking. QOL is a global concept based on abstract internal experiences, and self-assessments offer no support in interpreting and labeling inner experiences. More specific and concrete skills and abilities – such as knowledge of diagnosis, usage of coping strategies, social skills and conflict management - could have constituted more appropriate outcome measures.

### 4.2 Conclusions

This Internet-based intervention for adults with Autism spectrum disorder showed feasibility regarding adherence and participant satisfaction. However, the combination of the many themes in relation to the treatment length, a model heavily relying on own responsibility, the heterogeneity in the needs of autistic individuals and methodological limitations are likely explanations for the lack of effect in this trial. Further improvements in the treatment protocol are therefore warranted, and we suggest that future studies on IBI for ASD would benefit from more flexibility in content in accordance with the specific needs of the participants. The current study provides useful information for the development of future treatments for individuals with ASD.

## Funding

This work was supported by the 10.13039/501100009228Region Örebro Län, Sweden (grant numbers: OLL-935396, OLL-879651, OLL-887401, OLL-833131, OLL-785501, OLL-736321, OLL-878311).

## Declaration of competing interest

None.

## Appendices Supplementary data

Appendix A Table A.1 Treatment modules, strategies and excercises. Appendix A

Appendix B Table B.1 Questionnaire on self-perceived gains and development due to the treatment. Appendix B
