# Supplementary material for: An internet-based behavioral intervention for adults with autism spectrum disorder – A randomized controlled trial and feasibility study
Source: Internet Interv. 2023 Sep 19;34:100672. doi: 10.1016/j.invent.2023.100672 (PMC10523266; doi:10.1016/j.invent.2023.100672)
Supplement: Appendix A — Table A.1 Treatment modules, strategies and excercises. [file mmc1.docx]

| **Table A.1.** Treatment modules, strategies and excercises | | | |
| --- | --- | --- | --- |
| **Theme** | **Examples of strategies involved** | **Exercise** |  |
| 1. Introduction, diagnostic criteria | - | Reflect on own symptoms and treatment expectations. |  |
| 2. Behavioural change & individual goals | Setting goals according to the S.M.A.R.T^a^-model | Determine own goal |  |
| 3. Behavioural change | Functional analyses to understand how thoughts, feelings and behaviours interact | Conduct functional analyses of own behaviour |  |
| 4. Mentalization | Ask questions, ask for clarification, consult others. | Reflect on misunderstandings, interpret other people’s behaviour |  |
| 5. Social interaction I – Initiate & keep a conversation going | Active listening, ask questions, associating | Start a conversation |  |
| 6. Social interaction II – End a conversation, talking by phone | Use script in conversations, using of white lies when suitable | Practice phone calls |  |
| 7. Problem solving | Problem solving in 6 steps | Try a problem solving technique |  |
| 8. Emotions & non-verbal communication | Interpreting body language and nonverbal signals | Observe body language in a discussion |  |
| 9. Perception | Usage of sun glasses, ear buds, headphones, weight blanket, informing others of preferences | Reflect on own perceptual deviances |  |
| 10. Depression | Behavioural activation: identify and plan positive activities | Plan and carry through positively reinforced activities |  |
| 11. Social anxiety | Exposure, identify and challenge safety behaviours | Exposure to a social situation. |  |
| 12. Central coherence & logical errors | Question the plausibility of thoughts, consulting others | Reflect on experiences of logical errors and detail focus. Cognitive restructuring. |  |
| 13. Relations | Show appreciation, setting boundaries, honesty and trust | Reflect on emotions, friendship and love. Show appreciation to a loved one |  |
| 14. Stress and sleep | Specify what give and takes energy. Develop structure and routines around sleep, relaxation exercises | Plan and implement relaxation exercises |  |
| 15. Organization at home | Several strategies regarding planning and structure. | Try a new strategy to facilitate everyday activities |  |
| 16. Employment | Awareness of talents, personal traits and difficulties | Reflect on personal traits. Keep a journal of food intake and exercise (For next module) |  |
| 17. Diet & physical exercise | The plate model, regular meals | Evaluate the journal of food and exercise, and decide on something to change |  |
| 18. Summary | - | Evaluation |  |
| ^a^  Specific, Measurable, Action-Oriented, Realistic, and Time-bound | | |  |

**Appendix A**
