# Supplementary material for: An internet-based behavioral intervention for adults with autism spectrum disorder – A randomized controlled trial and feasibility study
Source: Internet Interv. 2023 Sep 19;34:100672. doi: 10.1016/j.invent.2023.100672 (PMC10523266; doi:10.1016/j.invent.2023.100672)
Supplement: Appendix B — Table B.1 Questionnaire on self-perceived gains and development due to the treatment. [file mmc2.docx]

| **Table B1.** Questionnaire on self-perceived gains and development due to the treatment | | | |
| --- | --- | --- | --- |
| Participating in MILAS have contributed to: | | | |
|  | Yes, definitely | To some extent | No |
| More knowledge about ASD |  |  |  |
| Increased self-awareness |  |  |  |
|  | | | |
| After MILAS I have: |  |  |  |
|  | Yes, definitely | To some extent | No |
| More social contacts than before |  |  |  |
| Greater understanding for my disability |  |  |  |
| Higher self-acceptance |  |  |  |
| Better express my needs |  |  |  |
| Increased well-being |  |  |  |

**Appendix B.**
